# Biological relapse in multiple myeloma: Outcome and treatment strategies in a Spanish real‐world setting

**DOI:** 10.1002/hem3.81

**Published:** 2024-07-04

**Authors:** Adrián Alegre, Mercedes Gironella, Fernando Escalante, Juan M. Bergua, Carmen Martínez‐Chamorro, Aurelio López, Esther González, Abelardo Bárez, Nieves Somolinos, Ernesto P. Persona, Alexia S. Cabrera, Alfons Soler, Belén I. Rodríguez, Joaquín M. López, Yolanda González, Verónica C. Giménez, Antonia Sampol, Carolina Muñoz, David Vilanova, Marta Durán, Carlos Fernández de Larrea, Spanish Myeloma Group (GEM_PETHEMA)

**Affiliations:** ^1^ Hospital Universitario de la Princesa Madrid Spain; ^2^ Hospital Universitari Vall d'Hebron Barcelona Spain; ^3^ Hospital Universitario de León, León Spain; ^4^ Hospital San Pedro de Alcántara Cáceres Spain; ^5^ Hospital Universitario Quirónsalud Madrid Spain; ^6^ Hospital Universitari Arnau de Vilanova Valencia Spain; ^7^ Hospital Universitario de Cabueñes Gijón Spain; ^8^ Hospital Nuestra Señora Sonsoles Ávila Spain; ^9^ Hospital Universitario de Getafe Getafe Spain; ^10^ Bioaraba [Onco‐Hematology Group], Vitoria‐Gasteiz, Osakidetza [OSI Araba], Hospital Universitario de Álava [Department of Hematology] Vitoria‐Gasteiz Spain; ^11^ Hospital Universitario de Gran Canaria Doctor Negrín, Las Palmas de Gran Canaria Spain; ^12^ Hospital Parc Taulí Sabadell Spain; ^13^ Hospital Clínico San Carlos Madrid Spain; ^14^ Hospital Universitario 12 de Octubre Madrid Spain; ^15^ Hospital Universitario Josep Trueta Girona Spain; ^16^ Hospital de Manises Valencia Spain; ^17^ Hospital Universitario Son Espases, Palma de Mallorca Spain; ^18^ Hospital Universitario Infanta Leonor Madrid Spain; ^19^ Celgene S.L. Unipersonal Madrid Spain; ^20^ Hospital Clínic i Provincial de Barcelona and IDIBAPS Barcelona Spain

## Abstract

Recommendations regarding the best time to start treatment in patients with relapsed/refractory multiple myeloma (RRMM) after biological relapse/progression (BR) are unclear. This observational, prospective, multicenter registry aimed to evaluate the impact on time to progression (TTP) of treatment initiation at BR versus at symptomatic clinical relapse (ClinR) based on the Spanish routine practice in adult patients with RRMM. Patients had two or less previous treatment lines and at least one previous partial response. Baseline characteristics and treatment outcomes were recorded, and survival was analyzed. Of 225 patients, 110 were treated at BR (TxBR group) and 115 at ClinR (TxClinR group) according to the investigators' criteria. The proportion of patients with higher ECOG, previous noncomplete remission (CR), and second relapse were significantly higher in the TxBR group compared to the TxClinR group. TheTxClinR group showed improved outcomes, including TTP, compared to the TxBR group. Progression‐free survival increased in the TxClinR group (56.2 months) compared to the TxBR group (32.5 months) (*p* = 0.0137), and median overall survival also increased (*p* = 0.0897). Median TTP was significantly longer in patients relapsing from a CR (50.4 months) and in their first relapse (38.7 months) compared to those relapsing from a non‐CR response (32.9 months) and in their second relapse (25.2 months). Physicians seemed to start treatment earlier in RRMM patients with poor prognosis features. Previous responses to anti‐MM treatment and the number of prior treatment lines were identified as prognosis factors, whereby relapse from CR and first relapse were associated with a longer time to progression.

## INTRODUCTION

Multiple myeloma (MM) is a malignant neoplasm characterized by an uncontrolled proliferation of a clone of plasma cells in the bone marrow, which can originate organ damage, including skeletal lesions, anemia, and renal failure.^1^, ^2^, ^3^ MM accounts for 1% of all malignant neoplasms and 10%–15% of all hematological malignancies, with a 5‐year prevalence of 450,579 cases.^4^ Data from 2020 indicated a global incidence of 176,404 cases, and a registered mortality of 117,077 deaths.^1^ The incidence is twice as high in the black population as in Caucasians, higher in men compared to women, and highest between the age of 60 and 70.^5^, ^6^ Although MM is known to be an age‐related disease, its etiology remains unknown.^1^, ^7^, ^8^, ^9^

Due to the clinical heterogeneity of MM, individualized MM treatments are recommended.^9^, ^10^, ^11^, ^12^ The different types of drugs with activity in MM are commonly prescribed in combined regimens in order to enhance their effects. At the time of the study design, the therapeutical arsenal included alkylating agents, corticosteroids, immunomodulatory drugs, and proteasome inhibitors. Autologous transplantation, maintenance, and consolidation are other treatment options within the therapy algorithm for patients with MM.^3^, ^13^ Despite the existing therapeutic arsenal, the natural evolution of the disease is progression and relapse, with only a fraction of patients achieving long‐term responses with significantly prolonged progression‐free survival.^3^, ^13^ Consequently, MM is characterized by relapses, which often occur after 3–4 years following initial diagnosis, with each subsequent response being of shorter duration.^3^, ^13^

One of the main factors to be considered when determining treatment for relapsed MM is whether the patient is symptomatic or asymptomatic. The International Myeloma Working Group (IMWG) protocol recommends starting treatment when patients develop symptomatic clinical relapse or progression (ClinR), a rapidly rising paraprotein level, or extramedullary disease.^14^ The same protocol states that asymptomatic patients with biological relapse (BR) showing a slow rise in paraprotein level can be managed with a watch‐and‐wait approach.^14^ After symptomatic ClinR, patients should be treated considering the duration of their response to previous therapy in addition to other parameters, such as age, performance status, comorbidities, and previous/residual treatment‐related toxicities.^14^, ^15^ These patients can be included in clinical trials for relapsed MM without problems. In most patients, BR will progress to ClinR within a median of approximately 5 months, even though a small percentage of patients (around 20%) may remain in BR without progression for several years.^16^, ^17^ Despite these recommendations, recent publications show that treatment initiation after BR may improve patient survival,^18^, ^19^ suggesting that early treatment would be the best option after BR.

Despite the importance of the timing of treatment in patients with BR, clear criteria regarding its initiation are still missing. The aim of this observational registry was to evaluate the clinical impact of starting the different anti‐MM regimens authorized in Spain during the study period after BR versus starting treatment at the time of ClinR in the routine clinical practice. This study hypothesized that early treatment after an asymptomatic relapse might result in better clinical outcomes with a longer time to progression compared to initiation of treatment after clinical relapse.

## MATERIALS AND METHODS

### Study design and participants

**Figure 1:**
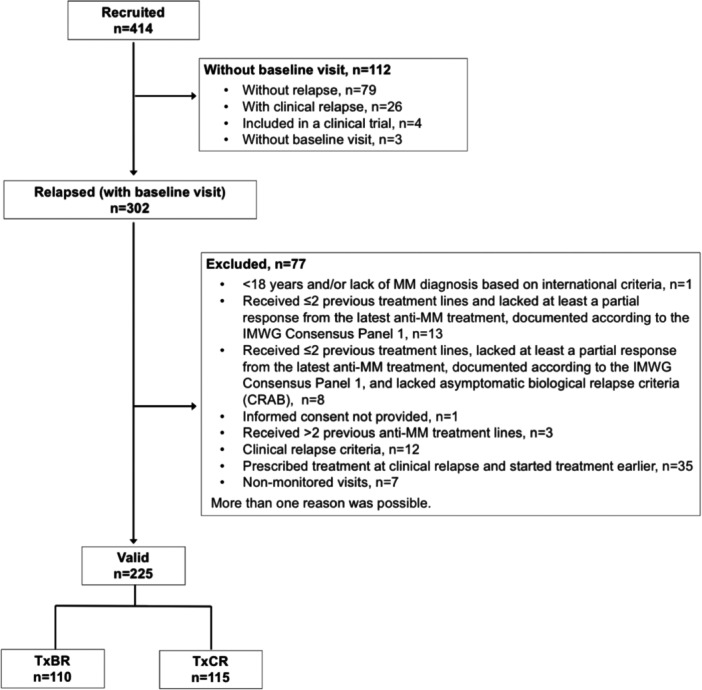
Study population diagram.

This was an observational, prospective, multicenter, single‐country registry designed to evaluate the decision of early therapy in asymptomatic relapsed MM in a real‐world setting. Adult patients (aged ≥18 years) who had received no more than two lines of treatment with at least one partial response to their latest MM treatment (documented according to IMW consensus panel 1)^20^ were recruited between May 2013 and December 2015 in 41 Spanish hospitals from the Spanish Myeloma Group (GEM‐PETHEMA). Patients diagnosed with first or second BR were initially recruited and, to increase the number of study patients, those in a pre‐BR phase were also recruited after study start (Figure 1); and when relapsing, patients were included in the study. Patients in a pre‐BR phase were defined as those followed up every 1–2 months with an appropriately documented lack of biological relapse within the 2 months (±15 days) previous to study inclusion. A complete list of inclusion and exclusion criteria is included in Table S1. Patients were classified into two groups according to the time of treatment initiation (BR vs. ClinR), which was chosen by the principal investigators in each center. The TxBR group included patients treated after BR (and prior to ClinR), and the TxClinR group included those treated after ClinR confirmation. Treatment allocation was based on the physicians' criteria according to the routine clinical practice. Patients were followed up during treatment according to the routine practice at each participating center and, after treatment finalization, patients were followed up every 6 months for up to 36 months. All patients signed a written informed consent at the time of enrollment. The study was conducted according to the Helsinki Declaration and the local Personal Data Protection Law (LOPD 15/1999).

The study protocol was approved by the Ethics Committee of Hospital Hospital Universitario de la Princesa (Madrid, Spain), and was endorsed by the Spanish PETHEMA group, and was registered on the clinicatrial.gov platform (NCT02394210).

### Variables and objectives

In addition to basic demographic variables, baseline clinical variables recorded were comorbidities, MM isotype (heavy and light chain), MM staging according to the International Staging System (ISS), patient's functional status according to the Eastern Cooperative Oncology Group (ECOG), presence of genetic abnormalities (high risk, standard risk, none), and previous lines of treatment. Additional baseline clinical variables included kappa and lambda light chain levels in the blood (mg/L), serum (g/dL), and urine M protein (mg/24 h) concentration, providing further insights into the disease's biochemical profile. Hemoglobin (g/dL), creatinine (mg/dL), macroglobulin β2 (mg/L), and serum albumin (g/dL) were also considered. The Durie‐Salmon staging system was also used to classify the severity of MM, complementing the ISS staging. Information on whether patients underwent transplantation was also recorded. Baseline treatment characteristics included previous treatments (first and second line) and responses. During the study, data from the antimyeloma therapeutic regimens administered were collected, including drug and treatment duration, in addition to response and date of relapse‐progression. An external scientific committee reviewed and agreed on the dates and categories of response for the study. Adverse events, including assessment of all second primary malignancies, were also reported and categorized according to the MedDRA dictionary (version 20.0).

The outcome of patients was described based on time‐dependent response parameters, according to the IMWG Consensus Panel 1 criterion.^20^ Time to progression (TTP) (primary objective) was defined as the time from asymptomatic BR to a new relapse or progression and time from treatment start to relapse or progression. Event‐free survival (EFS) was defined as the time between asymptomatic BR and the occurrence of an event, including relapse, progression, and death. Progression‐free survival (PFS) was measured as the time from asymptomatic BR to tumor progression or death. Overall Survival (OS) was defined as the time from asymptomatic BR to death.

### Statistical analysis

Quantitative variables were presented as the mean and standard deviation (SD), whereas categorical variables were presented as percentages. To evaluate the possible association between categorical variables, the Chi‐square test or Fisher's exact test were used. For one numerical and one categorical variable, descriptive statistics by groups were presented and, to evaluate their possible association, the *T*‐test, analysis of variance, or Wilhelm's tests and their nonparametric counterparts, the Wilcoxon or Kruskal–Wallis tests, were used. For the time‐dependent response parameters, a survival analysis by group (i.e., TxBR and TxClinR) was performed using the Kaplan–Meier estimator. Additional survival analyses according to previous complete response (Yes vs. No) and relapse number (First vs. Second) were performed. The median, Q1, and Q3 were presented with their 95% confidence interval (CI). The Log‐Rank test was used to assess the differences between groups. All statistical analyses were performed using the SAS® version 9.4 statistical package.

Data on the effectiveness of current treatments at the time of study initiation were used to calculate the sample size. The estimated total number of events needed was 277, assuming that the distribution of survival time is exponential in both groups. Sample size calculations were performed using nQuery v6.0 software (Janet D. Elashoff [2007] nQuery Advisor® Version 6.0.; Statistical Solutions).

### Data sharing statement

Data have not been deposited in any repository and will be made available by the corresponding author upon reasonable request.

## RESULTS

### Baseline demographic, clinical, and treatment characteristics of study patients

Of 414 patients with MM recruited, 302 experienced BR and had a baseline visit and, of those, 77 were excluded, resulting in a valid study population of 225 patients, 115 in the TxClinR group and 110 in the TxBR (Figure 1). The baseline demographic and clinical characteristics of the study patients are shown in Tables 1 and S2. Patients in the TxBR and TxClinR had similar characteristics regarding age, years for TxClinR and TxBR, sex, the isotype of MM, ISS classification, and the presence of genetic abnormalities. The treatment groups differed in other baseline parameters, including time from diagnosis to the baseline visit, number of previous treatments, comorbidities, and ECOG. Time from diagnosis was shorter in patients in the TxBR group compared to those in the TxClinR group. Even though the overall frequencies of patients with comorbidities were similar between groups (80.9% of patients in the TxClinR group and 84.5% of patients in the TxBR group), some specific comorbidities were present at different frequencies (Table S2). Mean ECOG values were significantly different between groups, with a worse performance status of patients in the TxBR group (*p* = 0.0468) (Table 1). Previous first‐ and second‐line MM treatments and responses according to treatment group are summarized in Table 2; Table S3 describes the previous treatment in detail.

**Table 1:** Baseline demographic and clinical characteristics of study patients, *N* = 225. (continued on next page)

	TxClinR (*n* = 115)^a^	TxBR (*n* = 110)^a^
**Demographic characteristics**		
Age (years), mean (SD)	69.5 (10.2)	68.5 (11.1)
	*n* = *115*	*n* = *109*
Sex, *n* (%)		
Male	60 (52.2)	53 (48.2)
Female	55 (47.8)	57 (51.8)
**Time between diagnosis and baseline visit (months), median (Q1, Q3)**	38.4 (25.3, 57.5)	29.3 (20.5, 56.8)
	*n* = *98*	*n* = *101*
Hemoglobin (g/dL), mean (SD)	13.2 (1.7)	13.0 (1.5)
Creatinine (mg/dL), mean (SD)	1.0 (0.5)	1.1 (0.7)
Microglobulin β2 (mg/L), mean (SD)	2.6 (1.5)	3.7 (3.2)
	*n* = *50*	*n* = 58
Serum albumin (g/dL), mean (SD)	4.2 (0.4)	4.5 (3.7)
	*n* = *102*	*n* = *98*
**Multiple myeloma characterization, *n* (%)**		
Multiple myeloma heavy chain isotype	*n* = *103*	*n* = *97*
Ig A	28 (24.3)	28 (25.5)
Ig G	74 (64.3)	67 (60.9)
Ig M	1 (0.9)	2 (1.8)
Multiple myeloma light‐chain isotype	*n* = *112*	*n* = *106*
Kappa	64 (55.7)	62 (56.4)
Lambda	48 (41.7)	44 (40.0)
Not available	3 (2.6)	4 (3.6)
ISS, *n* (%)	*n* = *89*	*n* = *87*
I	31 (27.0)	30 (27.3)
II	33 (28.7)	35 (31.8)
III	25 (21.7)	22 (20.0)
Kappa levels (mg/L), mean (SD)	46.9 (133.6)	73.5 (194.0)
	*n* = *63*	*n* = *70*
Lambda levels (mg/L), mean (SD)	25.2 (136.8)	46.5 (194.4)
	*n* = *63*	*n* = *71*
Serum M protein		
Detectable, *n* (%)	91 (79.1)	88 (80.0)
Levels (g/dL), mean (SD)	1.1 (1.0)	1.6 (1.1)
	*n* = *88*	*n* = *85*
Urine M Protein		
Detectable, *n* (%)	24 (20.9)	28 (25.5)
Levels (mg/24 h), mean (SD)	530.4 (1564.9)	483.1 (680.8)
	*n* = *22*	*n* = *28*
**ECOG at study enrolment, *n* (%)**	*n* = *64*	*n* = *51*
0	47 (73.4)	27 (52.9)
1	12 (18.8)	20 (39.2)
2	5 (7.8)	4 (7.8)
**Durie‐Salmon, *n* (%)**	*n* = *89*	*n* = *91*
IA	11 (12.4)	10 (11.0)
IB	1 (1.1)	2 (2.2)
IIA	21 (23.6)	32 (35.2)
IIB	4 (4.5)	1 (1.1)
IIIA	46 (51.7)	33 (36.2)
IIIB	6 (6.7)	13 (14.3)
**Genetic abnormalities at diagnosis, *n* (%)**	*n* = *105*	*n* = *106*
High risk^b^	8 (7.0)	10 (9.1)
Standard risk^c^	14 (12.2)	18 (16.4)
None	41 (35.7)	40 (36.4)
Not performed	52 (45.2)	42 (38.2)

Abbreviations: ECOG, Eastern Cooperative Oncology Group; ISS, International Staging System; SD, standard deviation; TxBR, treatment after biological relapse; TxClinR, treatment after clinical relapse.

^a^ Total number of patients per group; the number of patients with available data are indicated in italics in the corresponding cell.

^b^ *t*(4;14), *t*(14;16), del17p, 1q21 insertions, and 1p32 deletions.

^c^ Hyperdiploidy and *t*(11;14).

**Table 2:** Characteristics of previous multiple myeloma treatments, *N* = 225. (continued on next page)

	TxClinR	TxBR
**First‐line treatment**	*n* = 115^a^	*n* = 110^a^
Time between the start of treatment and the baseline visit (months), mean (SD)	44.1 (26.0)	41.5 (33.2)
	*n* = 105	*n* = 106
Induction, *n* (%)		
Yes	115 (100.0)	109 (99.1)
Recoded treatments, *n* (%)		
Scheme containing lenalidomide^b^	13 (11.3)	2 (1.8)
Regimen containing bortezomib^c^	84 (73.0)	67 (61.5)
Regimen containing thalidomide^d^	10 (8.7)	16 (14.7)
Others^e^	25 (21.7)	30 (27.5)
Autologous transplant, *n* (%)		
Yes	58 (50.4)	52 (47.3)
Consolidation, *n* (%)		
Yes	8 (7.0)	13 (11.8)
Maintenance, *n* (%)		
Yes	17 (14.8)	20 (18.2)
Maintenance treatments, *n* (%)		
Lenalidomide	7 (41.2)	6 (30.0)
Thalidomide	3 (17.6)	7 (35.0)
Bortezomib	2 (11.8)	7 (35.0)
Others	8 (47.1)	8 (40.0)
Best response obtained, *n* (%)		
Strict complete response (sCR)	26 (22.8)	8 (7.6)
Complete response (CR)	35 (30.7)	30 (28.6)
Very good partial response (VGPR)	35 (30.7)	38 (36.2)
≥Very good partial response (≥VGPR)	96 (84.2)	76 (72.4)
Partial response (PR)	18 (15.8)	29 (27.6)
Time between the best response and the baseline visit (months), mean (SD)	31.7 (24.4)	27.0 (27.7)
	*n* = 110	*n* = 100
Time between the end of treatment and the baseline visit (months), mean (SD)	30.6 (26.3)	22.1 (23.0)
	*n* = 106	*n* = 100
**Second‐line treatment**	*n* = 19	*n* = 32
Time between the start of treatment and the baseline visit (months), mean (SD)	39.2 (24.9)	28.6 (15.8)
	*n* = 17	*n* = 29
Induction, *n* (%)		
Yes	16 (84.2)	32 (100.0)
No	3 (15.8)	
Treatments^b^, *n* (%)		
LD	5 (31.3)	11 (34.4)
VD	7 (43.8)	6 (18.8)
V (bortezomib)		3 (9.4)
Others	5 (31.3)	16 (50.0)
Transplant, *n* (%)		
Yes	7 (41.2)	12 (37.5)
Consolidation, *n* (%)		
Yes	1 (5.9)	2 (6.3)
Maintenance, *n* (%)		
Yes	3 (17.6)	4 (12.5)
Maintenance treatments, *n* (%)		
Lenalidomide	1 (33.3)	1 (25.0)
Thalidomide	1 (33.3)	
Bortezomib		2 (50.0)
Others	2 (66.7)	2 (50.0)
Best response obtained, *n* (%)		
Strict complete response (sCR)	2 (11.8)	2 (6.3)
Complete response (CR)	4 (23.5)	7 (21.9)
Very good partial response (VGPR)	6 (35.3)	10 (31.3)
≥Very good partial response (VGPR)	12 (70.6)	19 (59.5)
Partial response (PR)	5 (29.4)	13 (40.6)
Time between the best response and the baseline visit (months), mean (SD)	30.6 (22.7)	17.6 (13.2)
	*n* = 17	*n* = 30
Time between the end of treatment and the baseline visit (months)	16.1 (20.8)	13.2 (16.5)
	*n* = 15	*n* = 29

Abbreviations: SD, standard deviation; TxBR, treatment after biological relapse; TxClinR, treatment after clinical relapse.

^a^ Total number of patients per group; the number of patients with available data are indicated in italics in the corresponding cell.

^b^ LD, lenalidomide, low dexamethasone dose; VD, bortezomib, dexamethasone; V, bortezomib, lenalidomide, dexamethasone; MPR, melphalan, prednisone, lenalidomide.

^c^ VTD, bortezomib, thalidomide, dexamethasone; MPV, melphalan, prednisone, bortezomib; MPT, melphalan, prednisone, thalidomide; VRD, bortezomib, lenalidomide, dexamethasone; PAD, bortezomib, doxorubicin, dexamethasone;

^d^ TD, thalidomide, dexamethasone; CTD, cyclophosphamide, thalidomide, dexamethasone; VTD, bortezomib, thalidomide, dexamethasone; MPT, melphalan, prednisone, thalidomide

^e^ CVAD, cyclophosphamide, vincristine, doxorubicin, dexamethasone; BP, bendamustine, prednisone.

Additionally, in the TxClinR group, we identified a population of 27 patients with BR who did not require treatment and did not progress to ClinR. These patients had a mean (SD) age of 72.1 (11.4) years and were included in the study (i.e., were in their first or second BR) a median (Q1, Q3) 49.2 (27.8, 79.2) months after diagnosis. Most had ISS I or II (80.0%), a small proportion had detectable M protein in the urine (21.0%), and none had high‐risk genetic abnormalities at diagnosis (Table S4).

Analysis of overall responses to previous MM treatments showed that the proportion of patients who relapsed from complete remission (CR) was significantly lower in the TxBR group (32.1%) compared to the TxClinR group (53.5%) (*p* = 0.0013) (Table 3). Furthermore, a higher proportion of patients in the TxBR group were in their second relapse (27.3%) compared to the TxClinR group (13.0%) (*p* = 0.0076) (Table 3). In addition to other baseline characteristics, the significant differences in responses to previous treatment and the number of previous lines between groups indicate that patients in the TxBR group had an overall worse prognosis compared to those in the TxClinR group.

**Table 3:** Characteristics of biological relapse at study enrolment, *N* = 225.

	TxClinR (*n* = 115)	TxBR (n = 110)	*p* Value
Patients relapsing from complete response, *n* (%)			
Yes	61 (53.5)	35 (32.1)	**0.0013**
No	53 (46.5)	74 (67.0)	
Prior lines, *n* (%)			
1	100 (87.0)	80 (72.7)	**0.0076**
>2	15 (13.0)	30 (27.3)	

Abbreviations: TxBR, treatment after biological relapse; TxClinR, treatment after clinical relapse.

### Treatment characteristics and outcomes

Table 3 summarizes the treatments for MM relapses received during the study and the responses obtained. Overall, patients in the TxClinR group had better responses compared to those in the TxBR group, with a higher complete response rate in the TxClinR group (25.0%) compared to the TxBR group (6.7%). Differences were also found between the percentages of stable disease, which were higher in the TxBR group (13.9%) with respect to the TxClinR group (34.3%) (Table 4).

**Table 4:** Study treatment characteristics and outcomes, *N* = 225.

	TxClinR (*n* = 115)	TxBR (*n* = 110)
Best response obtained, *n* (%)		
Strict complete response (SCR)	6 (8.3)	8 (7.6)
Complete response (CR)	18 (25.0)	7 (6.7)
Very good partial response (VGPR)	13 (18.1)	24 (22.9)
Partial response (PR)	23 (31.9)	26 (24.8)
Stable Disease	10 (13.9)	36 (34.3)
Relapse/progression	2 (2.8)	4 (3.8)
Progressive disease	1 (50.0)	2 (50.0)
Clinical relapse	1 (50.0)	2 (50.0)
Treatment overall response, *n* (%)		
Yes	60 (83.3)	65 (61.9)

Abbreviations: TxBR, treatment after biological relapse; TxClinR, treatment after clinical relapse.

### Survival analysis according to treatment group

**Figure 2:**
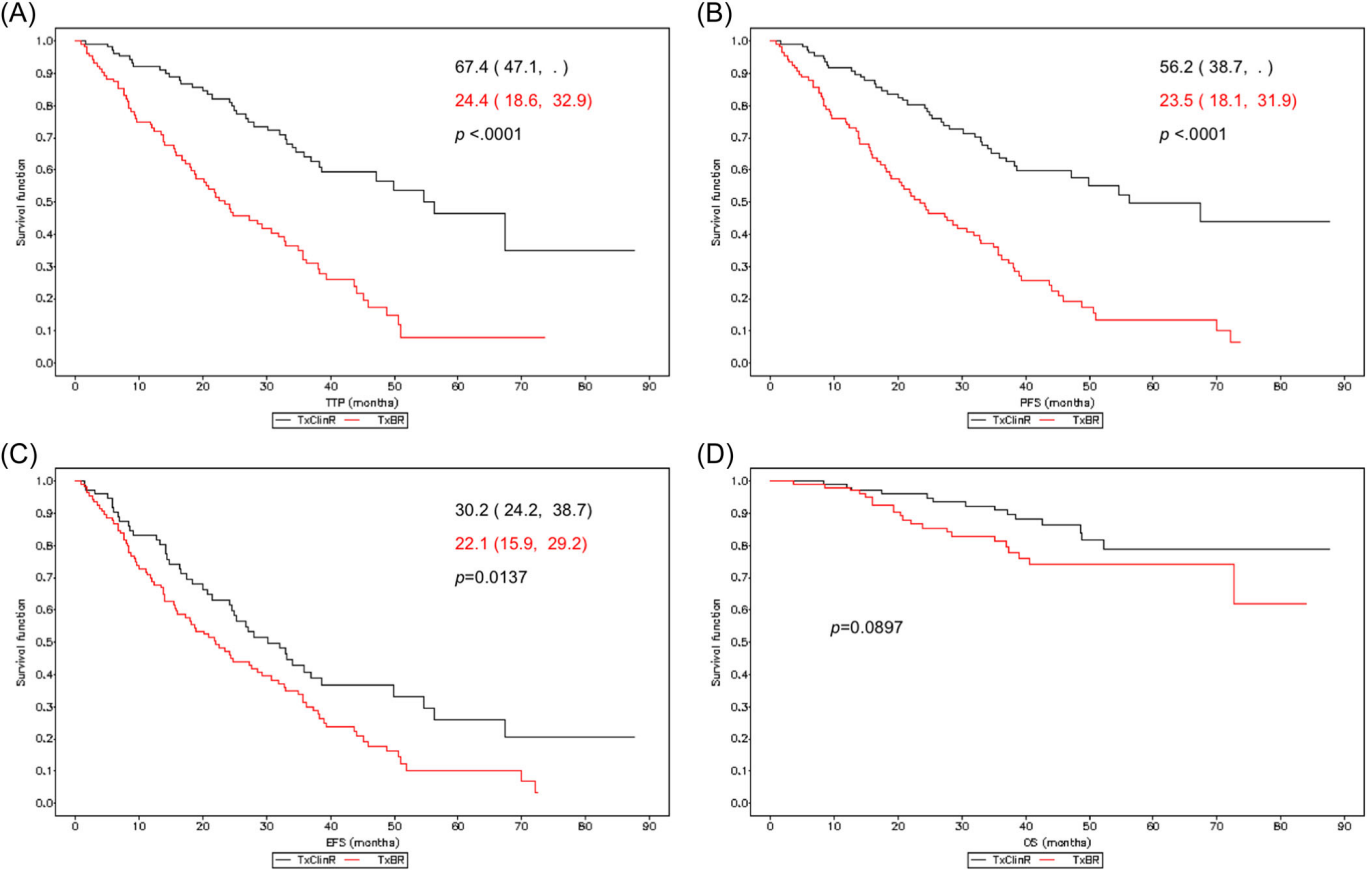
Time from biological relapse to progression (TTP) (A), progression‐free survival (PFS) (B), event‐free survival (EFS) (C), and overall survival (OS) (D) according to treatment group. Survival is presented as the median (95% confidence interval) (months); *p*‐values correspond to the Log‐Rank test for inter‐curve differences. Median overall survival in (D) could not be calculated due to the low number of events.

Survival analysis of time between BR and progression (TTP) (primary objective) showed significantly increased median survival time in the TxClinR (67.4 months) compared to the TxBR (24.4 months) group (*p* < 0.0001) (Figure 2A). With respect to progression‐free survival (PFS), survival curves showed a significant increase in the TxClinR group (56.2 vs. 32.5 months; *p* = 0.0137) (Figure 2B). Regarding EFS (i.e., the time between BR and ClinR/progression/death), median time was shorter in the TxBR group (22.1 months) compared to the TxClinR group (30.2 months; *p* < 0.0001) (Figure 2C). Patients in the TxClinR group also showed increased median overall survival (OS) compared to those in the TxBR group (*p* = 0.0897) (Figure 2D).

### Relationship between relapse characteristics and progression‐free survival

**Figure 3:**
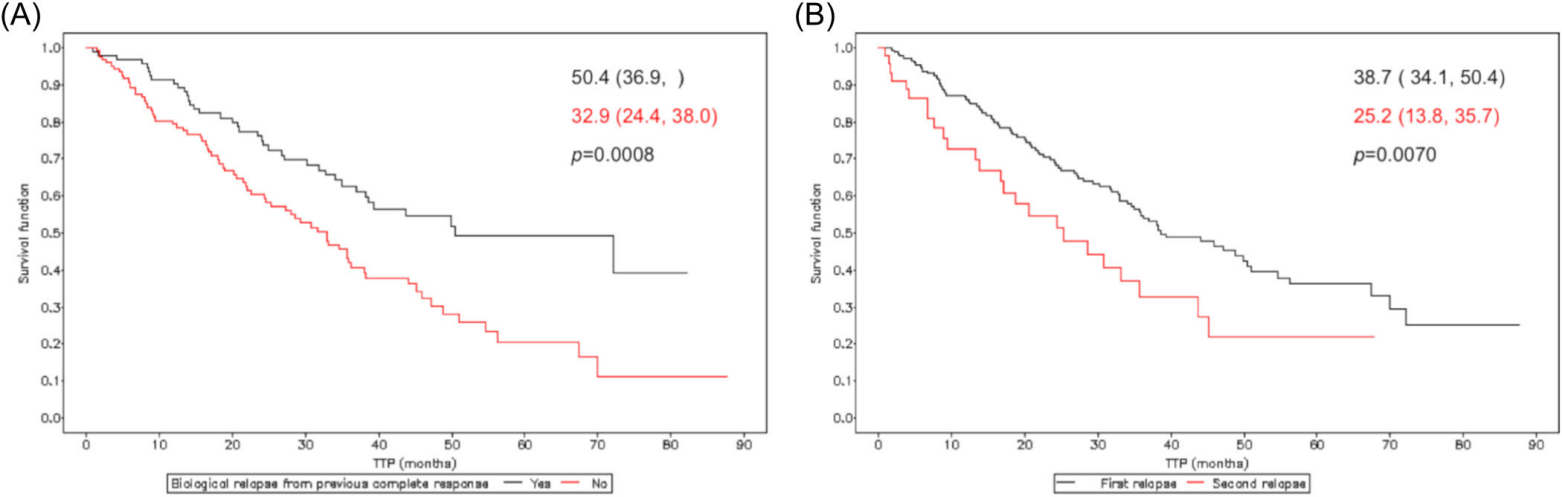
Time between biological relapse and progression according to previous response (A) and relapse number (B). Survival is presented as the median (95% confidence interval) (months); *p*‐values correspond to the Log‐Rank test for inter‐curve differences.

Given the differences between the TxBR and TxClinR groups regarding the proportion of patients relapsing from a complete response and the number of previous lines, the overall study population was classified according to these parameters since both factors are considered outcome predictors. Of 223 patients with available data, 96 patients previously experienced a complete response, and 127 lacked a previous complete response. Survival analysis of time from BR to progression according to these variables showed that patients who had achieved a previous complete response had increased median time to progression (50.4 vs. 32.9 months in patients with and without a previous complete response, respectively; *p* = 0.0008) (Figure 3A). Regarding relapse number, a total of 180 patients were in their first relapse and 45 in their second relapse at study enrolment. Patients in their second relapse showed a decreased time between BR and progression compared to those in their first relapse (25.2 vs. 38.7 months for patients in their second and first relapse, respectively; *p* = 0.0070) (Figure 3B).

### Safety outcomes

Table 5 summarizes the adverse events (AEs) experienced during the study. Based on observations, the absolute and relative number of patients with AEs was higher in the TxBR group than in the TxClinR group. Similarly, patients in the TxBR group presented grades 1–2 and 3–4 AEs at higher frequencies. Regarding treatment‐related AEs, the TxBR group had a higher frequency of these, causing up to twice as many temporary discontinuations compared to the TxClinR group.

**Table 5:** Summary of adverse events.

	TxClinR (*n* = 115)		TxBR (*n* = 110)	
	No. AE	No. patients (%)	No. AE	No. patients (%)
AE	831	78 (67.8)	1219	106 (96.4)
SAE	96	43 (37.4)	84	44 (40.0)
AE intensity 1–2	685	74 (64.3)	1010	102 (92.7)
AE intensity 3–4	131	48 (41.7)	201	64 (58.2)
AE intensity 5	15	12 (10.4)	8	8 (7.3)
AE related	243	56 (48.7)	409	81 (73.6)
AE dose modification	35	21 (18.3)	46	31 (28.2)
AE temporary suspension	46	23 (20.0)	121	44 (40.0)
AE definitive suspension	33	26 (22.6)	52	29 (26.4)

Abbreviations: AE, adverse events; SAE, serious adverse events; TxBR, treatment after biological relapse; TxClinR, treatment after clinical relapse.

## DISCUSSION

Results from this observational, prospective, multicenter study evaluating outcomes in patients with MM who received treatment after biological (TxBR group) or clinical relapse (TxClinR group) showed that treatment decision according to routine clinical practice criteria resulted in groups with different baseline clinical characteristics affecting aspects that determined prognosis. In addition to worse ECOG, a significantly higher proportion of patients in the TxBR group were in their second relapse and lacked a previous complete response compared to those in the TxClinR group. Survival analysis of time to progression according to these two factors showed, as expected, that patients after second MM relapse and relapsing from a noncomplete response had a significantly shorter time to progression compared to patients in their first relapse and relapsing from a complete response.

The study was designed to demonstrate the initial hypothesis, based on previous data, that treatment initiation after BR had a survival benefit for patients.^18^, ^19^ However, the results of the present study have shown the opposite, and patients in the TxBR group had a worse outcome, measured as TTP, PFS, EFS, and OS. Even though studies focused on the benefit of initiating treatment prior to ClinR are limited, results from the Endeavour trial showed increased survival when the treatment was initiated early in the course of the disease, before CRAB symptoms occurred.^18^ Similarly, a retrospective study comparing overall survival from start of first‐line therapy reported increased survival of patients starting second‐line treatment in biological relapse compared to those starting it at symptomatic relapse (125 vs. 81 months, respectively).^19^

The different baseline characteristics regarding relapse characteristics and, additionally, patient status between treatment groups may add an important bias for the correct analysis of this study, likely explaining the outcomes in this study. At the time of inclusion, the TxClinR group had a significantly higher proportion of patients who had achieved a complete response to previous treatments. Survival analysis showed that the time from BR to progression was longer in patients with a previous complete response. In this regard, numerous studies have reported that a previous complete response was associated with a longer PFS and, usually, a longer overall survival.^10^, ^16^, ^21^, ^22^, ^23^, ^24^, ^25^, ^26^, ^27^

In addition to previous response, another important baseline difference between groups was the number of previous lines of treatment: the TxBR group had a higher percentage of patients who were in their second relapse (13.0% vs. 27.3%; *p* = 0.0076). Current data on the natural history of MM indicates that the depth and duration of response following each relapse are generally diminished and that each subsequent remission has a shorter duration.^28^, ^29^, ^30^ Moreover, clinicians tend to treat earlier at subsequent relapses. Therefore, the population in the TxBR group, with a higher proportion of patients in their second relapse, would have an initial bias towards relapsing in a shorter time compared to the TxClinR group.

Another difference found in the basal parameters between the two populations was their physical status, measured using the ECOG. Several studies have shown that patients with MM with higher ECOG values have poorer clinical outcomes.^31^, ^32^, ^33^ Globally, the differences in time to progression together with the unbalanced baseline characteristics between groups indicate that patients in the TxBR group had worse predicted prognosis characteristics.

Results from the analysis of baseline characteristics showed a treatment selection bias. This bias indicates that, in the routine clinical practice, physicians tend to start treatment earlier (i.e., at BR) in patients with worse predicted prognosis characteristics and delay treatment start (i.e., until ClinR) in patients with a better prognosis. That highlights the accurate clinical decision taken by the principal investigators based on their patients' profile in this real‐world setting. The prolonged PFS after ClinR of patients in the TxClinR group (median 31.5 months) found in this study reflects the good prognosis for this patient group, further supporting the good clinical criteria of their physicians when deciding when to treat the patients.

Results from this study should be interpreted in the context of limitations associated with its real‐world setting. The major one was the criterion used for the inclusion of patients in each treatment group, based on physicians' decisions, which resulted in a treatment selection bias and unbalanced treatment groups. This limitation was associated with the real‐world setting of this study and precluded the appropriate analysis of the main objective, raising the need for future studies with a randomized design. Nevertheless, the different baseline characteristics of the TxClinR and TxBR groups yielded results that contradicted previous publications and prompted additional analyses, resulting in the identification of baseline relapse characteristics (i.e., previous lines and previous complete response) with a prognosis value. Another limitation of the study was associated with its size, which was smaller than planned. Even though a large number of patients from many different centers were initially recruited, many of them were not eligible, resulting in a smaller sample for analysis. The reduced valid sample size decreased statistical power, which may have impacted the results obtained from the statistical analyses.^34^, ^35^ It is also worth mentioning that treatment regimens available throughout the prolonged duration of this study (2013–2018) were different as new therapies were introduced more recently. Notably, emerging treatments such as bispecific antibodies and chimeric antigen receptor (CAR) T‐cell therapies are promising treatment options for RRMM and have altered the treatment landscape for patients with RRMM.^36^, ^37^, ^38^ However, bispecific antibodies are only available in the third‐line setting and beyond (after receiving an immunomodulatory agent, a proteasome inhibitor, and an anti‐CD38 antibody), which is not the scope of this study.^39^, ^40^, ^41^ CAR‐T cells were approved as advanced treatment based on clinical trial results, and have been recently approved in earlier treatment lines (cita cel in second line and ide cel in third line).^42^, ^43^ Therefore, patients in this study may have been candidates for these advanced treatments, particularly those with high‐risk BR or ClinR. Similarly, during the course of this study, new imaging tests have been introduced in the routine management of patients with RRMM, enabling an earlier symptomatic relapse detection, likely contributing to additional bias. Furthermore, in routine practice, physicians increasingly aim to avoid the complications of MM affecting the skeletal system by starting treatment earlier.

Even though the real‐world setting of this study precluded the reliable analysis of its primary objective, it reflected the results obtained in the routine practice in real‐life patients without strict selection criteria. The observed selection bias emerging from the criteria used by specialists in the real‐world practice of MM allowed us to identify predictive prognostic variables. To our knowledge, this is the first observational prospective study including this profile of patients.

In conclusion, in this study of Spanish routine clinical practice, physicians tend to start treatment earlier for MM after biological relapse/progression in those patients with worse predictive prognostic factors. Patients relapsing after only one line of treatment and those with previous complete remission show longer progression‐free survival compared to those in their second relapse and those relapsing from a noncomplete response, confirming the prognostic value of these factors.

## AUTHOR CONTRIBUTIONS

Adrián Alegre, Mercedes Gironella, and Carlos Fernández de Larrea contributed to study design and conception, data collection, and data interpretation. Fernando Escalante, Juan M. Bergua, Carmen Martínez‐Chamorro, Aurelio López, Esther González, Abelardo Bárez, Nieves Somolinos, Ernesto P. Persona, Alexia S. Cabrera, Alfons Soler, Belén I. Rodríguez, Joaquín M. López, Yolanda González, Verónica C. Giménez, Antonia Sampol, and Carolina Muñoz contributed to data collection. David Vilanova and Marta Durán contributed to data interpretation. All authors contributed to drafting the manuscript and revised and approved the final manuscript.

## CONFLICT OF INTEREST STATEMENT

Adrián Alegre received research support from Janssen, BMS‐Celgene, Amgen, Sanofi, GSK, Grifols, Pfizer, Abbvie, and Novartis, received honoraria for speakers bureaus from BMS‐Celgene, Amgen, GSK, Janssen, and Sanofi, and participated on a Scientific Advisory Boards for Janssen, BMS‐Celgene, Amgen, Sanofi, Oncopeptides, GSK, and Takeda. Mercedes Gironella received honoraria for lectures, presentations, speakers bureaus, manuscript writing, or educational events from BMS and Janssen. Fernando Escalante received honoraria for lectures, presentations, speakers bureaus, manuscript writing or educational events from Amgen, Janssen, and Sanofi, support for attending meetings and/or travel from GSK, Janssen, BeiGene, and BMS, and participated on a Data Safety Monitoring Board or Advisory Board for Takeda, Amgen, Janssen, Sanofi, BMS, BeiGene, and GSK. Juan M. Bergua received grants or contracts from Astellas Farma, received honoraria for lectures, presentations, speakers bureaus, manuscript writing or educational events from BMS, Janssen, and Jazz, and support for attending meetings and/or travel from Abbvie. Carmen Martínez‐Chamorro received honoraria for lectures, from Janssen, Abbvie, Amgen, and BMS, received support for attending meetings and/or travel from Janssen, and participated on a Data Safety Monitoring Board or Advisory Board for Janssen, BeiGene, and Abbvie. Ernesto P. Persona received honoraria for lectures, presentations, speakers bureaus, manuscript writing or educational events from Celgene and support for attending meetings and/or travel from Janssen. Alexia S. Cabrera participated in Advisory Boards for BMS‐Celgene, AstraZeneca, Amgen, Abbvie and received honoraria for lectures, presentations, speakers bureaus, manuscript writing, or educational events from Janssen, Amgen, Abbvie, AstraZeneca. Alfons Soler received honoraria for lectures, presentations, speakers bureaus, manuscript writing or educational events from Janssen‐Cilag, Celgene‐BMS, Abbvie, Astra Zeneca, and Roche, support for attending meetings and/or travel from Celgene‐BMS, Janssen‐Cilag, Abbvie, and Amgen, Belén I. Rodríguez received honoraria for lectures, presentations, speakers bureaus, manuscript writing or educational events from Tropos formación, Celgene, Janssen, and Sanofi, and support for attending meetings and/or travel from Celgene and Janssen. Joaquín M. López participated in advisory boards for BMS, Janssen, Roche, Gilead, and Novartis. David Vilanova and Marta Durán are BMS employees and stockholders. Aurelio López, Esther González, Abelardo Bárez, Nieves Somolinos, Yolanda González, Verónica C. Giménez, Antonia Sampol, Carolina Muñoz, and Carlos Fernández de Larrea declare no conflicts of interest.

## FUNDING

This study was funded by Celgene, a BMS company.

## Supporting information

Supporting information.

## Data Availability

The data that support the findings of this study are available from the corresponding author upon reasonable request.
