# Supplementary material for: Biological relapse in multiple myeloma: Outcome and treatment strategies in a Spanish real‐world setting
Source: Hemasphere. 2024 Jul 4;8(7):e81. doi: 10.1002/hem3.81 (PMC11223993; doi:10.1002/hem3.81)
Supplement: Supplementary file 1 — Supporting information. [file HEM3-8-e81-s001.docx]

# **Supplementary information for the manuscript “Biological Relapse in Multiple Myeloma: Outcome and Treatment Strategies in a Real-World Setting” by A Alegre et al.**

**Supplementary Tables**

**Table S1.** Inclusion and exclusion criteria

| Inclusion Criteria | Exclusion criteria |
| --- | --- |
| 1. Patients of both sexes, aged 18 years or older, diagnosed with MM based on internationally established criteria. 2. Patients with MM who have received no more than two lines of treatment and have achieved at least a partial response (≥RP) with their last anti-MM treatment, duly documented according to the IMW Consensus Panel 1^11^ criteria:    1. Patients who are not in relapse/non-symptomatic biological progression but who prior to the start of the study, the investigator decided to closely follow up on every one to two months, provided that there was a blood test (proteinogram and/or immunofixation) performed within two months (+/- 15 days) prior to inclusion showing that the patient was not in relapse or asymptomatic progression, or    2. Patients who presented criteria for relapse/non-symptomatic biological progression (without CRAB criteria), defined as an increase ≥25% from the lowest value reached during response, in any of the following parameters, and provided that there was a blood test (proteinogram and/or immunofixation) performed within two months (+/- 15 days) prior to inclusion showing that the patient was not in relapse or asymptomatic progression:       1. Serum M protein (absolute increase must be ≥0.5 g/dL) and/or       2. Urine M-component (absolute increase must be ≥ 200 mg/24 hours) and/or       3. Only in patients without measurable disease in serum and urine, the 25% increase from the lower level of the difference between related and unrelated FLC (absolute increase >10mg/dL).       4. In patients with unmeasurable M protein in serum and urine, and unmeasurable FLC levels, 25% increase in the percentage of plasma cells in bone marrow (absolute percentage must be ≥10%).       5. In patients with asymptomatic biological relapse since complete response, reappearance of M protein in serum or urine by immunofixation or electrophoresis, in two consecutive samples. 3. Patients who gave written consent after the nature and purpose of the study was clearly explained to them (written informed consent). | 1. Patients who participated in any interventional clinical trial. Inclusion of patients who were participating in other observational studies was allowed, at the discretion of the investigator. 2. Patients who refused to participate in the study. 3. Patients who have received more than two previous lines of anti-myeloma treatment. 4. Patients who, in the investigator's opinion, presented physical or mental incapacity to understand the information provided to them, and/or to answer the questions asked by their physician, in the framework of the study. 5. Criteria for relapse or clinical progression (symptomatic disease or organic lesions related to the disease), defined as:    1. Renal function impairment: serum creatinine ≥ 2 mg/dL or 177 mmol/L (new onset; if residual, it was considered if it had worsened as a result of myeloma).    2. Hypercalcemia (> 11.5 mg/dL; > 2.875 mmol/L).    3. Anemia: hemoglobin < 10 g/dL or 2 g/dL below normal values and provided that it was not attributable to a cause other than MM.    4. Bone lesion: new osteolytic lesions (with respect to diagnosis) observed in the 3 months prior to inclusion, current pathological fractures or increased osteopenia (with respect to diagnosis) in the radiological bone series.    5. Others: amyloidosis with current organ damage, recurrent bacterial infections (more than 2 episodes in 12 months), symptomatic hyperviscosity, presence of plasmacytomas. |

**Table S2.** Description of comorbidities at the time of enrolment according to treatment group, n (%)

|  | **TxClinR n=115^a^** | **TxBR n=110^a^** |
| --- | --- | --- |
| **Patients with comorbidities** | 93 (80.9) | 93 (84.5) |
| **Comorbidities at the time of enrolment, *n (%)*** |  |  |
| Infections and infestations | 2 (1.7) | 6 (5.5) |
| Benign neoplasms, malignant and unspecified | 6 (5.2) | 9 (8.2) |
| Medical and surgical procedures | 4 (3.5) | 9 (8.2) |
| Cardiac disorders | 15 (13.0) | 10 (9.1) |
| Atrial fibrillation | 7 (6.1) | 5 (4.5) |
| Blood and lymphatic system disorders | 11 (9.6) | 10 (9.1) |
| Anemia | 7 (6.1) | 3 (2.7) |
| Thrombocytopenia | 4 (3.5) | 6 (5.5) |
| Reproductive system and breast disorders | 10 (8.7) | 12 (10.9) |
| Benign prostatic hyperplasia | 9 (7.8) | 9 (8.2) |
| Metabolism and nutrition disorders | 39 (33.9) | 41 (37.3) |
| Diabetes mellitus | 6 (5.2) | 7 (6.4) |
| Type 2 diabetes mellitus | 12 (10.4) | 8 (7.3) |
| Dyslipidemia | 13 (11.3) | 16 (14.5) |
| Hypercholesterolemia | 5 (4.3) | 11 (10.0) |
| Immune system disorders | 5 (4.3) | 6 (5.5) |
| Nervous system disorders | 15 (13.0) | 11 (10.0) |
| Peripheral neuropathy | 6 (5.2) | 4 (3.6) |
| Endocrine disorders | 6 (5.2) | 17 (15.5) |
| Hypothyroidism | 1 (0.9) | 12 (10.9) |
| Gastrointestinal disorders | 11 (9.6) | 16 (14.5) |
| Hiatal hernia | 5 (4.3) | 8 (7.3) |
| Musculoskeletal and connective tissue disorders | 28 (24.3) | 24 (21.8) |
| Back pain | 6 (5.2) | 3 (2.7) |
| Osteoarthritis | 6 (5.2) | 2 (1.8) |
| Osteoporosis | 4 (3.5) | 10 (9.1) |
| Psychiatric disorders | 7 (6.1) | 6 (5.5) |
| Renal and urinary disorders | 9 (7.8) | 14 (12.7) |
| Chronic kidney disease | 6 (5.2) | 4 (3.6) |
| Respiratory, thoracic and mediastinal disorders | 10 (8.7) | 7 (6.4) |
| Vascular disorders | 48 (41.7) | 47 (42.7) |
| Hypertension | 45 (39.1) | 43 (39.1) |

| **Table S3.** Description of previous multiple myeloma treatments according to treatment group, N=225 | | |  |
| --- | --- | --- | --- |
|  | **TxClinR**  **n=115^a^** | **TxBR**  **n=110^a^** | |
| **First-line treatments, *n (%)*** |  |  | |
| VTD | 9 (7.8) | 14 (12.8) | |
| VD | 42 (36.5) | 26 (23.9) | |
| LD | 11 (9.6) | 2 (1.8) | |
| VRD | 2 (1.7) |  | |
| MPV | 36 (31.3) | 41 (37.6) | |
| MPT | 1 (0.9) | 2 (1.8) | |
| MPR | 0 (0.0) | 0 (0.0) | |
| TD | 1 (0.9) | 1 (0.9) | |
| PAD | 5 (4.3) | 4 (3.7) | |
| CVAD | 1 (0.9) |  | |
| CTD | 0 (0.0) | 0 (0.0) | |
| BP | 0 (0.0) | 0 (0.0) | |
| Others | 24 (20.9) | 29 (26.6) | |
| Autologus Transplant  **Second-line treatments, *n (%)*** | 58(50.4%)  n=19 | 52 (47.3%)  n=32 | |
| LD | 5 (31.3) | 11 (34.4) | |
| VD | 7 (43.8) | 6 (18.8) | |
| V |  | 3 (9.4) | |
| Others | 5 (31.3) | 16 (50.0) | |
| Abbreviations: TxBR, treatment after biological relapse; TxClinR, treatment after clinical relapse; SD, standard deviation; VTD, bortezomib, thalidomide, dexamethasone; VD, bortezomib, dexamethasone; LD, lenalidomide, low dexamethasone dose; VRD, bortezomib, lenalidomide, dexamethasone; MPV, melphalan, prednisone, bortezomib; MPT, melphalan, prednisone, thalidomide; MPR, melphalan, prednisone, lenalidomide; TD, thalidomide, dexamethasone; PAD, bortezomib, doxorubicin, dexamethasone; CVAD, cyclophosphamide, vincristine, doxorubicin, dexamethasone; CTD, cyclophosphamide, thalidomide, dexamethasone; BP, bendamustine, prednisone. ^a^Total number of patients per group; the number of patients with available data are indicated in italics in the corresponding cell. | | |  |

| **Table S4.** Baseline demographic and clinical characteristics of study patients in whom treatment was not initiated and who did not require treatment | |
| --- | --- |
|  | **TxClinR n=27^a^** |
| Demographic characteristics |  |
| Age (years), *mean (SD)* | 72.1 (11.4) |
| Sex, *n (%)* |  |
| Male | 12 (44.4) |
| Female | 15 (55.6) |
| Time between diagnosis and baseline visit (months), *median (Q1, Q3)* n=23 | 49.2 (27.8, 79.2) |
| Hemoglobin (g/L), *mean (SD)* | 12.9 (1.7) |
| Creatinine (mg/L), *mean (SD)* | 1.2 (0.6) |
| Microglobulin ß2 (mg/L), *mean (SD)* n=15 | 2.8 (1.6) |
| Serum albumin (g/dL), *mean (SD)* n=26 | 4.0 (0.6) |
| Kappa (mg/L), *mean (SD)* n=13 | 26.6 (76.3) |
| Lambda (mg/L), *mean (SD)* n=13 | 6.3 (11.8) |
| Serum M protein |  |
| Detectable n=26 | 21 (80.8) |
| (g/dL), *mean (SD)* n=20 | 0.7 (0.5) |
| Urine M protein *n=19* |  |
| Detectable | 4 (21.0) |
| (g/dL), *mean (SD)* n=4 | 266.3 (175.5) |
| Multiple myeloma characterization, n (%) n=25 |  |
| Multiple myeloma heavy chain isotype |  |
| Ig A | 9 (36.0) |
| Ig G | 16 (64.0) |
| Ig M | 0 (0.0) |
| Multiple myeloma light-chain isotype |  |
| Kappa | 17 (63.0) |
| Lambda | 10 (37.0) |
| ISS, *n (%)* n=20 |  |
| I | 9 (45.0) |
| II | 7 (35.0) |
| III | 4 (20.0) |
| Durie-Salmon, *n (%)* n=23 |  |
| IA | 4 (17.4) |
| IB | 1 (4.3) |
| IIA | 7 (30.4) |
| IIB | 0 (4.5) |
| IIIA | 8 (34.8) |
| IIIB | 3 (13.0) |
| Genetic abnormalities at diagnosis, *n (%)* n=15 |  |
| High risk^b^ | 0 (0.0) |
| Standard risk^c^ | 4 (26.7) |
| None | 11 (73.3) |
| Transplant, n (%) | 14 (51.9) |
| Abbreviations: ECOG, Eastern Cooperative Oncology Group; ISS, International Staging System; TxBR, treatment after biological relapse; TxClinR, treatment after clinical relapse; SD, standard deviation.  ^a^Unless otherwise indicated  ^b^t(4;14), t(14;16), del17p, 1q21 insertions, and 1p32 deletions ^c^Hyperdiploidy and t(11;14) | |
